# Plasma-Treated Nanostructured Resistive Gas Sensors: A Review

**DOI:** 10.3390/s25072307

**Published:** 2025-04-05

**Authors:** Mahmoud Torkamani Cheriani, Ali Mirzaei

**Affiliations:** Department of Materials Science and Engineering, Shiraz University of Technology, Shiraz 71557-13876, Iran; m.torkamani@sutech.ac.ir

**Keywords:** plasma treatment, toxic gas, gas sensor, sensing mechanism

## Abstract

Resistive gas sensors are among the most widely used sensors for the detection of various gases. In this type of gas sensor, the gas sensing capability is linked to the surface properties of the sensing layer, and accordingly, modification of the sensing surface is of importance to improve the sensing output. Plasma treatment is a promising way to modify the surface properties of gas sensors, mainly by changing the amounts of oxygen ions, which have a central role in gas sensing reactions. In this review paper, we focus on the role of plasma treatment in the gas sensing features of resistive gas sensors. After an introduction to air pollution, toxic gases, and resistive gas sensors, the main concepts regarding plasma are presented. Then, the impact of plasma treatment on the sensing characteristics of various sensing materials is discussed. As the gas sensing field is an interdisciplinary field, we believe that the present review paper will be of significant interest to researchers with various backgrounds who are working on gas sensors.

## 1. Introduction to Toxic Gases

**Figure 1:**
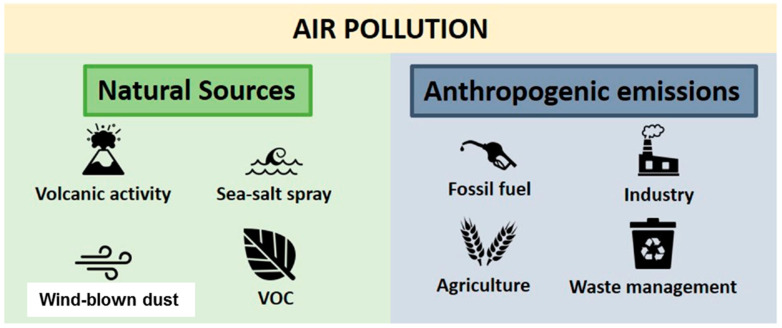
Sources of air pollution [2]. With permission from MDPI.

Air pollution is due to the existence of unwanted substances in the air, affecting its cleanness and quality. It is a serious issue in most countries and led to 4.14 million premature deaths worldwide in 2019 [1]. In addition to particulate matter, toxic gases are among the main components of polluted air. NO_2_, SO_x_, O_3_, and CO gases are among the most dangerous gases often found in polluted air. Natural air pollution sources include volcano eruptions and wind-blown dust, and anthropogenic sources include the burning of fossil fuels, agricultural activities, waste management, and so on (Figure 1) [2].

Air pollution has many negative effects on animals [3] as well as on the environment [4]. For example, NO_x_ and SO_2_ gases in polluted air can directly affect photosynthesis and bring about premature leaf senescence, eventually decreasing crop yields [5,6]. In addition, it has detrimental effects on human health. Air pollutants may influence influenza transmission [7], intensify COVID-19 mortality [8], induce respiratory diseases [9], such as bronchoconstriction [10], asthma [11], and lung cancer [12], affect pregnancies and related things such as low birth weight, preterm birth, and fetal hyperinsulinism [13], cause cardiovascular diseases [14,15], and impact the immune system [16]. Since about 90% of people live in places with polluted air [17], it is necessary to monitor the air quality precisely. In this regard, the development of reliable gas sensors is vital.

## 2. Resistive Gas Sensors: An Introduction

There are various types of gas sensors that can be used to detect toxic gases. The most common gas sensors are optical [18], electrochemical [19], surface acoustic wave [20], and resistive [21]. Resistive sensors have high response, high stability, swift dynamics, ease of design and fabrication, compact size, and low price. They are realized from semiconducting materials, and currently, semiconducting metal oxides are widely used for this purpose. However, metal oxide gas sensors have some drawbacks, such as poor selectivity, high sensing temperature, and humidity interference [22]. Thus, recently, other semiconductors, such as carbon-based materials, including carbon nanotubes [23], graphene [24], reduced oxide graphene [25], conducting polymers (CPs) [26], transition metal dichalcogenides (TMDs) [27], and MXenes, have been employed for the fabrication of resistive gas sensors in order to reduce sensing temperature and increase the selectivity to a specific gas.

**Figure 2:**
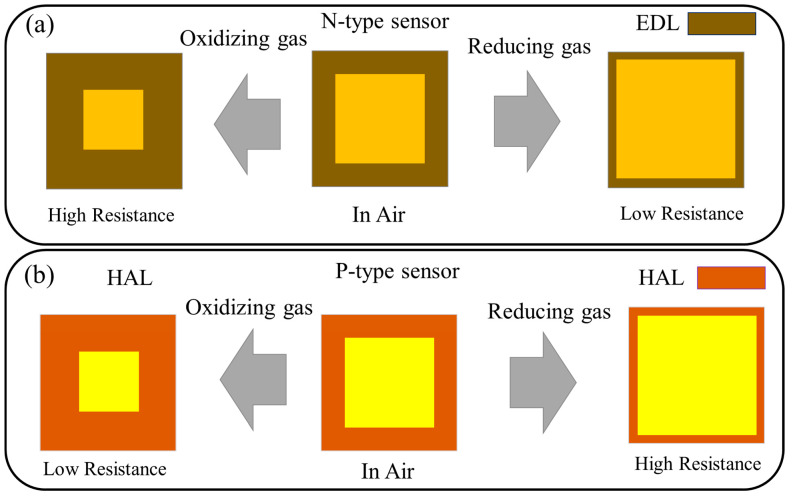
Basic gas sensing mechanism of resistive gas sensors: (**a**) n- and (**b**) p-type sensors.

In resistive gas sensors, the sensing layer is applied on the surface of an insulating substrate, such as alumina, which is equipped with electrodes. Also, sometimes a micro-heater is attached to a substrate to provide sufficient heat to increase the sensing device to the desired temperature [28]. The principle of the gas sensing mechanisms of resistive sensors is based on a variation in resistance in the presence of target gas. In general, there are two types of semiconducting materials based on the majority of charge carriers. In n- and p-type gas sensors, electrons and holes are the main charge carriers, respectively. When a resistive gas sensor is exposed to air, oxygen gas will be adsorbed on it, and thanks to its high electrophilic nature, it takes electrons from the sensor surface. Accordingly, on n-type gas sensors, a so-called electron depletion layer (EDL) will appear in which the concentration of electrons is lower relative to the inner part. Hence, the resistance of n-type gas sensors increases in air relative to vacuum conditions. Furthermore, a hole accumulation layer (HAL) will appear on the surface of p-type gas sensors in which the concentrations of holes are higher than those in the core part of the sensor. When an n-type sensor is put in a reducing gas atmosphere, the gas reacts with adsorbed oxygen on the sensor surface, releasing electrons back to the sensor. Hence, the thickness of the EDL decreases, bringing about a decrease in sensor resistance. Upon exposure to oxidizing gas, the gas takes further electrons from the sensor surface, leading to the expansion of the EDL and an increase in sensor resistance (Figure 2a). For a p-type sensor, the release of electrons in the presence of an n-type gas leads to the narrowing of the HAL and an increase in sensor resistance, whereas in an oxidizing gas atmosphere, the further abstraction of electrons leads to an expansion of the HAL and a decrease in resistance [29,30,31] (Figure 2b).

Currently, the main challenges of nanostructured resistive gas sensors are as follows: (i) the development of highly sensitive gas sensors with the capability of gas sensing down to ppt level; (ii) the development of highly selective gas sensors; (iii) the development of humidity-resistant gas sensors; and (iv) the development of gas sensors with low power consumption. To address the above-mentioned challenges, the synthesis of sensing materials with high surface areas, the combination of various sensing materials, functionalization with appropriate noble metals, functionalization using plasma treatment, UV illumination, and operation of the sensors in self-heating mode have been proposed.

In more detail, there are various techniques to boost the sensing performance of resistive sensors. Heterojunction formation [32], doping [33], noble metal decoration [34], morphology engineering [35], UV illumination [36], high-energy irradiation [37], and plasma exposure are among the most widely used techniques. In particular, some problems of resistive gas sensors, namely selectivity and high operating temperature, can be at least partially addressed by plasma treatment. Plasma exposure causes a change in the amount of oxygen vacancies, thereby facilitating the adsorption of oxygen gas on the surface of the plasma-treated sensor. Since oxygen species engage in sensing reactions, better gas sensing properties can be obtained. Furthermore, by the right selection of plasma type, other species such as –F, -C can be added on the surface of the sensor, acting as favorable adsorption sites for target gas molecules. Hence, both selectivity and working temperature can be improved.

So far, there have been no published review papers dealing with the effect of plasma treatment on the gas sensing properties of resistive sensors. Hence, in this review paper, we will review the effect of plasma treatment on the sensing features of resistance sensors.

## 3. The Plasma Concept

When energy is supplied to a gas, its temperature gradually increases, and by the further provision of energy, the kinetic energy of gas molecules significantly increases, leading to the collision of more gas molecules. Hence, electrons and ions are formed in the gas, leading to the existence of an electrical charge in the gas. This state of matter is known as plasma, originating from Greek, which means ‘something molded’, indicating a glowing gas which alters its shape based on the container. Gases are often electrical insulators, while plasmas have an equal amount of positive and negative charge carriers along with neutral particles, giving them electrical conductivity [38].

In equilibrium plasma, local thermodynamic equilibrium exists among the plasma species and collision processes, where heavy particles and electrons will be at almost identical temperatures. In contrast, non-equilibrium plasma or cold plasma involves a thermodynamic imbalance among the electrons and heavy particles, and the temperature of the heavy particles is much lower than that of electrons. During cold plasma production, heating the entire gas stream (air or individual gases like Ar and He) is undesirable; thus, energy is directed to the electrons via the electrical discharge in the gas [39]. Corona discharge, dielectric barrier discharge (DBD), and cold plasma jet are among the most common ways to generate cold plasma. To generate cold plasma at atmospheric pressure, a high voltage is applied for the generation of a gas discharge, and the discharge easily proceeds to arc discharge. Furthermore, energy should be selectively transferred to electrons using effective methods without raising the temperature of the gas [38].

Cold plasma treatment is an environmentally friendly technique without the production of toxic waste, and thanks to its operation under atmospheric pressure, it is an appropriate technique for the treatment of low-melting-point or heat-sensitive materials and substrates [40]. In particular, flexible polymeric substrates have low surface energy and poor wettability. Therefore, the adhesion between electrodes and a polymeric substrate is weak. Accordingly, plasma surface modification of polymeric substances can overcome this shortage [41].

In both corona discharge and DBD, the sample to be plasma treated is put between electrodes in a fixed space under atmospheric pressure. In corona discharge, by applying a DC electrical source in a pulsed mode, a lighting crown is built out of many streamers, while in DBD, a high-frequency source is employed for this purpose. During corona discharge, the cathode is a conductive wire, and the anode is the sample. A DBD reactor usually has two parallel metal electrodes at a fixed distance covered with a dielectric material, and the sample is placed between them. The formed plasma has many homogeneously distributed micro-streamers across the electrodes [42].

Plasma treatment is a flexible, fast, green, and non-contaminating method of changing surface morphology and composition. Compared to conventional routes, this method is faster and needs fewer reagents. Also, only the surface area is affected by the plasma treatment, without affecting the bulk region. By optimizing the plasma parameters, including plasma power, exposure time, and the type of gas, various functional groups with different amounts can be added on the surface of the host material [43]. Furthermore, plasma treatment can be performed at atmospheric pressure, facilitating large-scale treatment for mass production [44]. Moreover, plasma can be employed to deposit thin layers over the substrate in a process called plasma spray [45,46]. In the following sections, we will discuss the impact of plasma treatment on the gas sensing properties of resistive gas sensors.

## 4. Plasma-Treated Gas Sensors

### 4.1. Plasma-Treated Carbon Nanotube Gas Sensors

Carbon nanotubes (CNTs) are one-dimensional materials with high conductivity, a large surface area, and the possibility of functionality [47,48]. Nevertheless, homogeneous dispersion of CNTs is a challenge owing to the presence of attractive Van der Waals forces among CNTs, leading to agglomeration and weak solubility in most solvents. Hence, it is required to change the surface properties of CNTs via surface treatment or chemical functionalization [49]. Compared to the surface treatment of CNTs using strong acids such as HNO_3_ and H_2_SO_4_, which is time-consuming and dangerous, plasma treatment using oxygen is a simple, clean, and effective way to functionalize CNTs. Acid treatment leads to the presence of carboxylic acids, ethers, and so on on the surface of CNTs, while oxygen plasma treatment increases the number of oxygen-bearing defects on the entire surface of the CNTs. Also, the hydrophilic nature of CNTs can be boosted thanks to the presence of oxygen-containing species on the surface of CNTs. Furthermore, the bulk features of CNTs remains untouched during plasm treatment, without any structural destruction [50]. As a result of oxygen plasma treatment, oxygenated vacancies and functional groups will be present on the surface of CNTs, which are very reactive species and act as favorable adsorption sites for gas molecules [51]. Therefore, plasma treatment has been extensively applied on CNTs to increase their gas sensing performance [52,53,54,55,56].

In this regard, Bannov et al. [57] functionalized the surface of multi-walled CNTs (MWCNTs) using oxygen plasma exposure followed by C_2_H_2_(CO)_2_O plasma treatment. The MWCNTs were comprised of intertwined MWCNTs with a mean diameter of 20–50 nm. After plasma treatment, most of the MWCNTs were strongly etched by oxygen plasma, and only a few MWCNT bundles remained. Based on an XPS study, the plasma treatment led to the presence of a high amount of oxygen-containing surface groups on the MWCNTs. The sensor resistance was increased after the plasma treatment due to the oxidation of the MWCNTs and the loss of the connections among MWCNT networks. At room temperature (RT), the response of the fabricated sensor to 500 ppm NH_3_ was only 11.7%, while after plasma treatment it increased to 31.4%, demonstrating the promising role of plasma treatment. The increase in sensor response was related to the enhanced NH_3_ adsorption by the oxygen-rich surfaces as a result of the plasma treatment. Due to the reducing nature of NH_3_ gas, it should react with adsorbed oxygen to release the electrons on the sensor surface. Hence, the higher amounts of oxygen species on the sensor surface as a result of plasma exposure led to a higher probability of the reaction with NH_3_, resulting in a higher response. The same group [58] investigated the effect of oxygen plasma exposure time (3, 5, and 7 min) on the NH_3_ gas sensing properties of MWCNTs. The sensor exposed to oxygen plasma for 5 min exhibited the highest response to NH_3_ gas, which was related to the presence of the highest amount of adsorbed oxygen species on the surface of CNTs. In another study, Dong et al. [59] studied the effect of various plasma types using Ar, O_2_, CF_4_, and SF_6_ gases on the gas sensing properties of single-walled CNTs (SWCNTs). Thanks to reactive ion etching, defects were generated on the SWCNTs. Based on a Raman analysis, the intensity of the D-band to G-band (I_D_/I_G_) ratio of the pristine sample was only 0.14, while after plasma treatment by the above-mentioned gases, it was changed to 0.23, 0.36, 0.33, and 0.5, respectively. Therefore, more defects were generated on the surface of the plasma-treated samples. The pristine sensor not only showed a very low response to both NO_2_ and NH_3_ gases, but also the recovery time was very long (more than 20 min). Also, the resistance did not completely return to its initial value. In contrast, the plasma-treated sensors showed better sensing performance. The sensor treated with O_2_ plasma revealed a higher response to NO_2_ gas thanks to the presence of defect sites and adsorbed oxygen species groups, which led to the better adsorption and reaction of NO_2_ gas on the sensor surface. Also, the responses of the sensors treated with CF_4_ and SF_6_ were higher than NH_3_ gas relative to other gases, which was attributed to the sufficient adsorption energies and easy charge transfer between the NH_3_ and C–F bonds (CF_4_ and SF_6_) of the plasma-treated MWCNTs.

**Figure 3:**
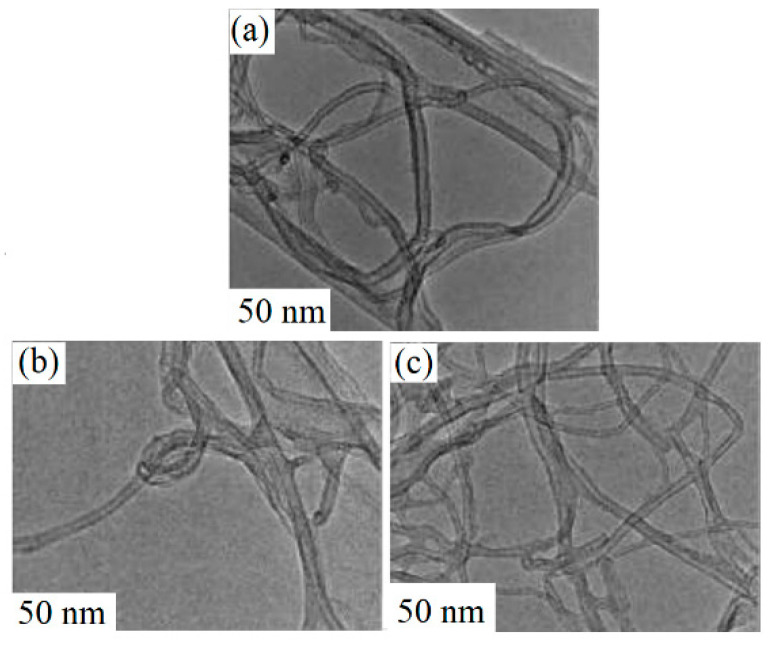
TEM views of MWCNTs: (**a**) pristine, (**b**) He-, and (**c**) Ar-treated MWCNTs [60]. With permission from Elsevier. Copyright (2020).

Santosh et al. [60] used Ar and oxygen plasma treatment using a fixed 100 sccm of gas for 3 min on MWCNTs for improvement of the gas sensing capacity. The sensor treated with Ar plasma revealed a higher response to other gas sensors, which was related to the greater extent of the surface modifications by the Ar plasma. At 65 °C, the maximum response to ethanol gas was observed with a response [(R_a_ – R_g_)/R_a_] of 1.7 to 100 ppm ethanol. Argon is much heavier than He, and hence, more defects were generated on the MWCNTs after Ar treatment. The diameter of the MWCNTs decreased after plasma exposure due to the etching effect of plasma (Figure 3a–c). In addition, based on Raman analysis, the amorphous wrinkled layer on the pristine sensor was removed after plasma treatment, which eventually improved the crystalline behavior of the MWCNTs. Furthermore, thanks to the higher crystallinity and high amount of carbon defects, the conductivity increased after plasma exposure and enhanced the interaction of the MWCNTs with ethanol. Finally, the plasma treatment led to the formation of dangling bonds, which acted as favorable sites for ethanol gas adsorption.

Ham et al. [61] modified MWCNTs by oxygen plasma for 10–50 s. The morphology of the MWCNTs did not change in up to 20 s of plasma exposure. However, by increasing the treatment time to more than 30 s, the surface became highly rough, and the MWCNTs were partially etched. The sensor treated with plasma for 20 s showed a higher response to NH_3_ gas relative to other gas sensors, indicating that the sensitivity of the MWCNT gas sensors can be enhanced through defect generation and the adding of oxygenated functional groups. The enhanced sensitivity was ascribed to the generation of hydrogen bonds between NH_3_ gas and surface oxygen groups on the MWCNTs.

The sensing performance of nitrogen-plasma-treated SWCNT gas sensors has rarely been investigated. In this regard, Zamansky et al. [62] synthesized SWCNTs via a CVD method and then used nitrogen plasma to modify their surfaces. Based on characterization results, the defects were introduced on SWCNTs after plasma treatment. In particular, with longer plasma exposure, the amount of substitutional N defects relative to -NH_2_ surface groups increased, indicating the incorporation of N into the SWCNT lattice. Also, due to the exposure of the MWCNTs to air after plasma treatment, many oxygen-containing defects were detected. The pristine sensor was almost insensitive to the gases. In contrast, the sensor treated for 19 min exhibited a response of 121% to 50 ppm NO_2_, and its response to 50 ppm NH_3_ was 36% at RT. The enhanced performance was related to the existence of oxygen- and nitrogen-related defects, which served as desirable adsorption sites for gas molecules. Also, the amount of metallic SWCNTs with poor gas sensing properties decreased after etching. Based on DFT calculations, the gas adsorption on defect sites was more favorable compared to basal plane sites. In addition, while NH_3_ sensing was accelerated by hydrogen bond formation with surface groups such as COOH, the adsorption of NO_2_ was mainly caused by the oxidation of carbon defect regions and physisorption.

Ham et al. [63] investigated the impact of plasma treatment on the NH_3_ sensing properties of SWCNTs with different amounts of semiconducting SWCNTs (66 and 90 wt.%). After oxygen plasma treatment, the sensor with 66 wt.% semiconducting SWCNTs exhibited a 5.5-times increase in sensitivity relative to the pristine sensor, while the sensor with 90 wt.% semiconducting SWCNTs revealed a 13-times increase in sensitivity compared to the pristine sensor. The pristine SWCNT sensor revealed a very long response time and incomplete recovery, while the plasma-treated sensors showed much quicker dynamics, with complete recovery of baseline resistance after treatment. Based on an XPS study, the amount of oxygen functional groups was significantly improved after plasma treatment. The NH_3_ molecules formed strong hydrogen bonds with oxygen ions on the oxidized SWCNTs. Therefore, a significant response improvement was observed. Also, in the sample with 90 wt.% semiconducting SWCNTs, the sp^3^/sp^2^ ratio increased from 0.256 to 0.611 after the plasma treatment, indicating that the higher semiconducting nature of plasma-treated SWCNTs and the existence of sp^3^ defects provided favorable adsorption sites for NH_3_ gas.

**Figure 4:**
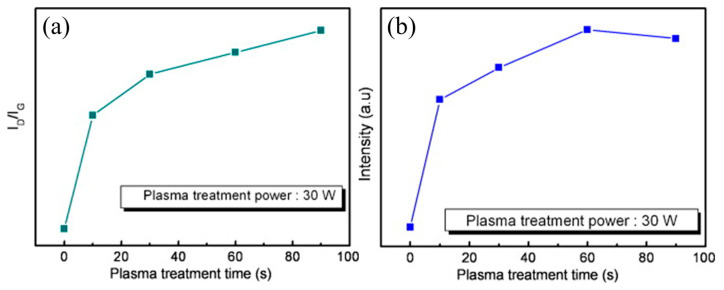
(**a**) Changes in the I_D_/I_G_ and (**b**) oxygen vacancy of SWCNTs as a function of plasma treatment time [67]. With permission from Elsevier. Copyright (2009).

CPs with high conductance, flexibility, simple synthesis procedures, and low cost are among the most promising materials for RT gas sensing applications [64,65]. Hence, composite formation between CNTs and CPs is a favorable strategy for RT gas sensing, while plasma exposure can further increase their performance [66]. In this regard, Yoo et al. [67] studied the effect of oxygen plasma treatment (10, 30, 60, and 90 s) on the NH_3_ sensing capability of an MWCNT–polyaniline (PANI) composite. Based on a Raman analysis, the number of defects on the MWCNTs increased with the increase in plasma time (Figure 4a). During plasma exposure, oxygen ions destroyed the structure of the MWCNTs by turning them into carbon particles and amorphous carbon, along with the creation of more defects relative to the pristine MWCNTs. Based on an XPS study, the concentration of surface oxygen increased with the increase in the plasma exposure time up to 60 s and then decreased by a longer treatment of 90 s, which was attributed to chemical etching of the MWCNTs (Figure 4b). This resulted in the thinning or bending of the MWCNTs. At RT, the response of the plasma-treated MWCNTs was three times that of the pristine sensor thanks to the formation of hydrogen bonds between polar NH_3_ and oxygen-containing defects on the MWCNTs. Also, the plasma-treated MWCNT-PANI composite sensor revealed a higher response to the plasma-treated MWCNTs thanks to the presence of PANI with a high intrinsic response to NH_3_ gas. The NH_3_ molecules abstracted protons from the PANI, forming energetically more favorable NH_4_^+^ ions, while the PANI changed into its base form with a different conductivity, resulting in the generation of a high sensing signal.

During the synthesis of CNTs, some impurities and contaminants are introduced into the CNTs. Even though purification procedures can be used, sometimes they are detrimental to the gas sensing properties of CNTs. In this regard, Kim et al. [68] investigated the impact of thermal annealing (T > 300 °C) and plasma treatment with oxygen on the characteristics and NH_3_ gas sensing properties of CNTs. The pristine SWCNTs had a hydrophobic nature with a water contact angle (WCA) of 84.91°. The thermally treated SWCNTs again showed a hydrophobic nature with a WCA of 79.07°, while the plasma-treated SWCNTs showed a WCA of only 5.15°, indicating an increase in the hydrophobic nature after plasma treatment due to the adding of oxygen surface groups on the SWCNTs. The plasma-treated SWCNTs showed a decrease in sp^2^ bonding with an increase in sp^3^ bonding, indicating a change in electrical conductivity. Among the three sensors, the plasma-treated SWNT sensor exhibited the highest response and the fastest response time to NH_3_ gas. In addition, both the pristine and thermally treated SWNT sensors exhibited incomplete recovery of their resistance. While thermal cleaning of the SWCNTs removed impurities from the surface of the SWCNTs, the plasma treatment included cleaning and functionalization of the SWNTs at the same time to a greater extent, resulting in better sensing capability after plasma treatment.

Zhao et al. [69] applied plasma on CNTs for CO gas sensing. While the pristine CNTs showed no response to this gas, the plasma-treated CNTs were able to detect down to 5 ppm CO at RT. The improved sensing response was related to the conversion of metallic CNTs to semiconducting CNTs after plasma treatment, along with the promising effect of surface oxygen addition for sensing reactions with CO gas.

### 4.2. Plasma-Treated Graphene and Graphene Oxide Gas Sensors

Pristine graphene (G) has a high surface area and high conductivity, demonstrating its potential for sensing applications [70]. However, it generally has poor selectivity since the gas adsorption on G is based on Van der Waals interactions with gases, which limit its selectivity [24]. To address this issue, plasma treatment can be used [71]. In this regard, Masterapa et al. [72] applied plasma treatment on CVD-grown G for 5, 10, 20, and 30 s. The sample treated with plasma for 30 s revealed higher amounts of defects, as demonstrated by a Raman analysis, and hence it showed a higher response to NO_2_ and NH_3_ gases relative to other sensors. However, the response time of all the sensors was very long (10 min), and the recovery curves were not shown possibly due to very long recovery times.

Fluorination surface treatment could change the intrinsic properties of G. In this regard, Zhang et al. [73] synthesized monolayer fluorinated graphene (FG) by a SF_6_ plasma treatment (5–90 s). The concentration of F in the samples increased with the increase in plasma treatment time up to 20 s, and then it decreased. During the plasma treatment, the fluorine atoms attached to carbon atoms to form C−F bonds on the surface of G. After a critical time, the F atoms broke down some previously formed C−F bonds, and hence, F atoms were released from the surface of G. The pristine G sensor exhibited slow dynamics, and even after 500 s of recovery, only ∼66.7% of the initial resistance was recovered. The sensor treated with plasma for 20 s exhibited a response of 3.8% to 100 ppm NH_3_ gas at RT, with complete recovery of baseline resistance in less than 200 s. Based on DFT results, the improved performance was ascribed to the opening up of the band gap after fluorination and the enhanced adsorption of NH_3_ in the presence of surface functional groups.

Chung et al. [74] synthesized G films using the CVD route, and they were then treated with plasma ozone for 60, 70, 80, and 90 s. Among the fabricated sensors, the sensor with the ozone treatment time of 70 s showed a response of 19.7% to 10 ppm NO_2_ gas at RT, which was two times higher than that of the pristine G sensor. Also, the sensor was able to detect as low as 200 ppb NO_2_ gas. Further increasing the plasma treatment time resulted in a decrease in the sensing response due to extensive oxidation of G with high baseline resistance. The presence of sufficient amounts of oxygen groups on the surface of G resulted in an increase in adsorption sites and sensing reactions with the NO_2_ gas. However, the sensors showed long dynamics, and all sensors showed a long recovery time of ~20 min or longer.

CO_2_ is the main gas responsible for the greenhouse effect [75]. Hence, the development of sensitive CO_2_ sensors is crucial for environmental and industrial applications. Casanova-Chafer et al. [76] synthesized a G-CsPbBr_3_ nanocomposite and subsequently applied oxygen plasma treatment on it. The sensor exposed for 5 min to oxygen plasma exhibited a 3-fold improvement in gas sensing compared to the pristine sensor, with a limit of detection of 6.9 ppm. The improved sensing performance was attributed to the promising role of oxygen species, facilitating sensing reactions with CO_2_ gas on the sensor surface.

Graphene oxide is the oxidized form of G with two key advantages. First, the synthesis route of GO is easy using graphite as raw material, and hence its large-scale production is feasible. Second, in contrast to G, GO exhibits good hydrophilicity, making it possible to prepare stable aqueous colloids [77,78]. Nonetheless, the main shortcoming of GO is its high resistance, making it unsuitable for sensing applications [79]. In this regard, plasma treatment is a promising technique, allowing the reduction of GO by removing oxygen atoms during plasma exposure, without disrupting the GO lattice. Hydrogen or hydrogen-containing plasma with mild treatment conditions is an efficient and alternative route to the complex procedures for GO reduction. The hydrogen plasma contains radicals and atoms, which provide energy for the dissociation of oxygen functional groups. It effectively removes the oxygen functional groups at the edge sites and both basal planes while restoring C=C bonds [80]. After the reduction of GO, it becomes converted to reduced graphene oxide (rGO) with high conductivity, high amounts of surface defects, and also some oxygen surface groups along with a high surface area, all making it a good choice for gas sensing applications.

**Figure 5:**
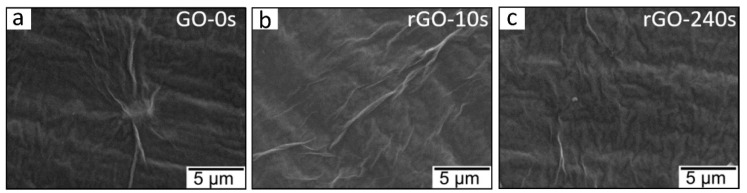
Surface morphology of (**a**) pristine GO and GO after plasma treatment for (**b**) 10 and (**c**) 240 s. With permission from Elsevier. Copyright (2024).

Hamzaj et al. [81] used hydrogen plasma treatment for 10, 20, 40, 120, and 240 s on GO to reduce it for gas sensing applications. The surface morphology of pristine GO showed some mild wrinkles, and it was not changed after plasma treatment for 10 and 240 s (Figure 5a–c). It should be noted that generally, plasma treatment does not significantly change the surface morphology.

**Figure 6:**
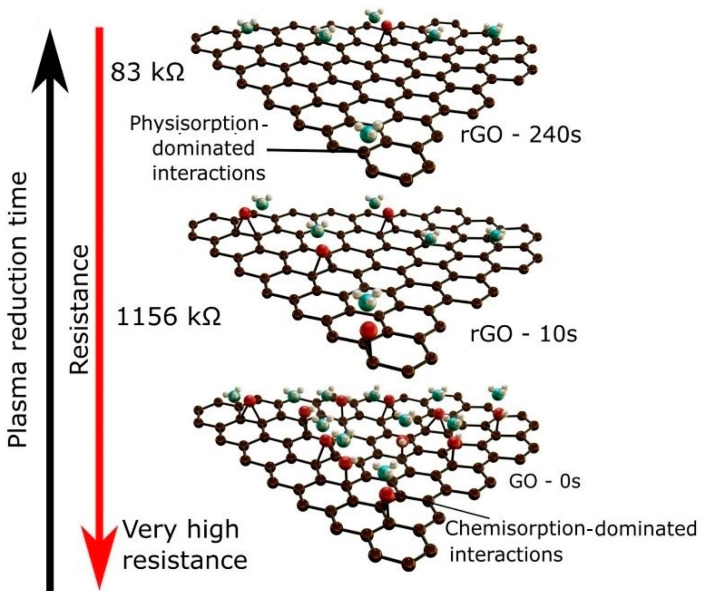
A possible physisorption/chemisorption-assisted sensing mechanism towards ammonia in plasma rGO sensors [81]. With permission from Elsevier. Copyright (2024).

Based on resistance measurement studies, the resistance gradually decreased with the increase in hydrogen plasma treatment (Figure 6), which effectively removed the oxygen groups from GO and partially restored the sp^2^-bounded carbon network, resulting in enhanced conductivity. In addition, during plasma exposure, the amorphous phases were etched, which contributed to the improved conductance. Based on various characterizations, the pristine GO had an oxygen content of 30 at.%, and after plasma treatment for 240 s, it decreased to 20 at%, confirming the reducing effect of plasma exposure on GO.

Among the different gas sensors, the sensor exposed to plasma for 20 s revealed the highest response to NH_3_ gas at RT. After 20 s of plasma exposure, the oxygen groups were not extensively removed, and hence, they provided sufficient channels for the physisorption of NH_3_. Meanwhile, chemisorption of NH_3_ was facilitated due to the presence of oxygen groups. This led to achieving the optimal sensing performance by providing a balance between both the chemisorption and physisorption phenomena.

### 4.3. Plasma-Treated ZnO Gas Sensors

The response of ZnO sensors also can be remarkably increased by plasma treatment [82,83,84]. In this context, Hou et al. [85] prepared ZnO thin films by sol–gel spin-coating deposition. Then, they were treated with O_2_ plasma for 3, 5, 8, 11, and 15 min. During plasma treatment for 3 and 5 min, the crystallinity increased thanks to the decrease in oxygen vacancies, while a further increase in plasma exposure time led to a decrease in the crystallinity due to the formation of zinc vacancies. Also, the roughness of the pristine ZnO thin film was 5.5 nm, which decreased to 3.6 nm after plasma treatment and then increased to 4.3 and 5 nm with further increase in the treatment time to 8 and 11 min, respectively. The sensor that underwent 8 min plasma exposure revealed a higher response of 65% to 50 ppm NH_3_ at RT compared to the other sensors. It manifested a higher baseline resistance relative to the pristine sensor, and hence, more reactions occurred between the adsorbed oxygen and NH_3_ gas, contributing to a higher sensing response.

**Figure 7:**
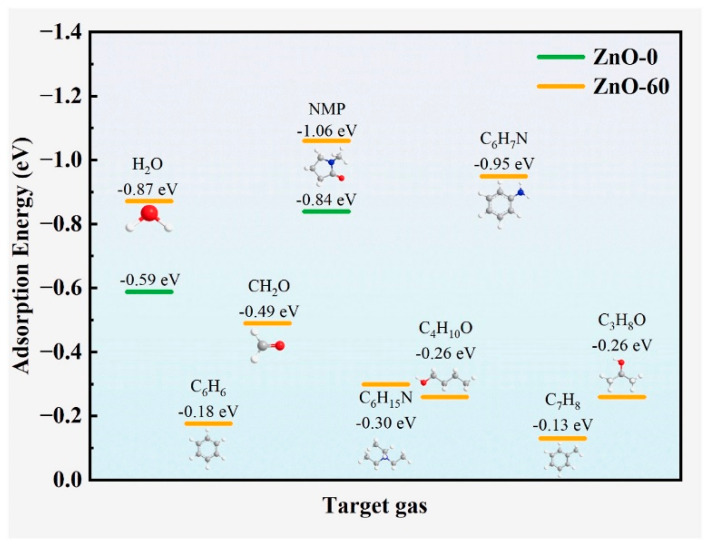
Adsorption energies of different gases on pristine and plasma-exposed (60 s) ZnO NRs [86]. With permission from Elsevier. Copyright (2024).

Gui et al. [86] produced ZnO nanorods (NRs) with average diameters of 300 nm on ceramic tubes by an in situ hydrothermal growth method at 140 °C. Then, they were exposed to oxygen plasma for 30, 60, and 90 s. Upon plasma exposure, not only did the surface become rough, but also the content of oxygen vacancies increased up to a plasma exposure time of 60 s. The sensor exposed to plasma for 60 s manifested a high response of 198 to 100 ppm N-methyl pyrrolidone (NMP) at 210 °C, which was three times higher than that of the pristine sensor. The improved performance originated from the presence of the highest amount of oxygen vacancies, which acted as highly active sites for oxygen adsorption, and the subsequent increase in reactions with target gas molecules. Based on DFT calculations, the adsorption energy of NMP on the oxygen-plasma-treated ZnO was higher than that of the pristine ZnO. Furthermore, the adsorption energy of NMP on ZnO was the largest (−1.06 eV) compared to other gases, leading to better selectivity to NMP gas (Figure 7).

Although the chemical solution method is widely used for sensing film deposition, it still suffers from poor adhesion between the film and substrate along with poor reproducibility. In this regard, atomic layer deposition (ALD) is a highly reliable method of film deposition, allowing precise control of the thickness of the film by adjusting the number of ALD cycles. Furthermore, it can be used for the deposition of uniform sensing layers on a substrate with high reproducibility [87,88]. In this regard, Li et al. [89] deposited ultrathin ZnO films (20 nm) by the ALD technique followed by Ar plasma treatment for 1, 5, and 10 min. Among the different samples, the one exposed to plasma for 5 min exhibited the highest amount of oxygen vacancies, as confirmed by XPS and EPR analyses. It revealed a maximum response of 21.6 to 100 ppm TEA at 250 °C with a low limit of detection of 22 ppb. The high selectivity to TEA was ascribed to the presence of active C–N bonds in TEA and the high electron-denoting properties of TEA. Furthermore, oxygen vacancies acted as electron donors and decreased the band gap of ZnO, eventually facilitating the adsorption and activation of TEA.

### 4.4. Plasma-Treated SnO_2_ Gas Sensors

SnO_2_ is among the most widely used sensing materials, thanks to its high stability, high mobility of electrons, ease of the synthesis, low price, nontoxicity, and abundance [90,91]. Plasma treatment has been used on SnO_2_ to modify its sensing properties [92,93]. Srivastava et al. [94] are among the leading researchers reporting the enhanced gas sensing properties of SnO_2_ sensors under oxygen and hydrogen plasma exposure. The sensitivity of a sensor treated with oxygen plasma was found to be about 10 times more than that of the pristine sensor, while in the case of hydrogen plasma, the response of the plasma-treated (15 min) sensor was seven times higher than that of the pristine sensor. Also, the same group [95] reported the enhanced gas sensing response of elemental-doped SnO_2_ gas sensors.

**Figure 8:**
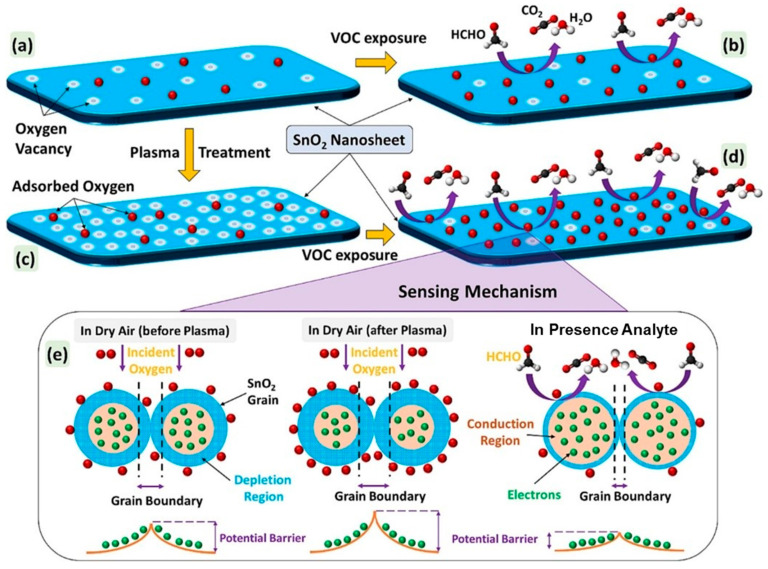
Schematic illustration of sensing mechanism of SnO_2_ NSs to VOCs: (**a**,**b**) pristine SnO_2_ NSs; (**c**,**d**) plasma-treated SnO_2_ NSs; (**e**) modulation of double Schottky barrier in the presence of plasma and VOC [96]. With permission from Elsevier. Copyright (2024).

Acharyya et al. [96] synthesized SnO_2_ nanosheets (NSs) through a hydrothermal route at 200 °C for 12 h. Then, the synthesized materials were exposed to Ar plasma for 2, 4, 7, and 10 min. At 270 °C, the sensor treated with Ar for 7 min revealed the highest response of 25 to 10 ppm HCHO gas. Also, the smaller molecule size and lowest activation energy of the HCHO compared to other gases accounted for the selective response to gas. The content of oxygen vacancies was highest in the sensor exposed to plasma for 7 min. This caused more oxygen and HCHO gas adsorption on the surface of the SnO_2_ NSs, leading to a higher response relative to the pristine sensor (Figure 8a–d). Furthermore, as indicated in Figure 8e, the SnO_2_-SnO_2_ homojunctions were formed in air, and the height of barriers was lower relative to that of the plasma-exposed sensor. Hence, in the presence of HCHO gas, the significant reduction in homojunction height in the case of the plasma-treated sensor led to the generation of a higher sensing response relative to the pristine sensor.

**Figure 9:**
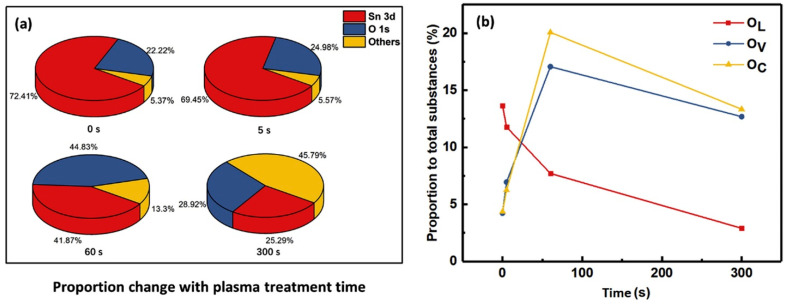
(**a**,**b**) The amounts of different species on Pd-SnO_2_ NFs versus plasma exposure time [99]. With permission from Elsevier. Copyright (2020).

Pd is a good catalyst for H_2_ gas dissociation, and therefore it is widely used as a decoration on the surface of resistive gas sensors [97,98]. Hu et al. [99] synthesized Pd-decorated SnO_2_ nanofibers (NFs) via electrospinning of SnO_2_ NFs followed by the decoration of Pd NPs using sputtering. Then, the samples were exposed to Ar plasma treatment for 5, 60, and 300 s. Based on an XPS analysis, the content of oxygen vacancies and adsorbed oxygen species increased after plasma treatment. The Sn-O bonds in the SnO_2_ dissociated during the collision with Ar ions, resulting in the generation of oxygen vacancies. Furthermore, the dissociated oxygen was chemisorbed on the oxygen vacancy sites. The sensor exposed to plasma for 60 s revealed the highest response of 53 to 500 ppm H_2_ gas at 130 °C. Figure 9a,b show the amounts of different enlacements as a function of plasma exposure time. The sensor exposed to plasma for 60 s exhibited the highest amount of oxygen vacancy and adsorbed oxygen species, both of which were highly beneficial for H_2_ gas sensing. However, the extension of plasma exposure to 300 s led to the degradation of sensing performance due to the decrease in both oxygen vacancy and adsorbed oxygen species. Also, the catalytic effect of Pd towards H_2_ dissociation was effective on the high sensing response towards H_2_ gas.

Chaturvedi et al. [100] used plasma treatment on Pd-doped SnO_2_ gas sensors. The synthesized materials were exposed to O_2_, H_2_, N_2_, and Ar plasma for 15 min. In all cases, the plasma-treated sensors revealed a higher response to CCl_4_, CO, LPG, C_3_H_7_OH, N_2_O, and CH_4_ gases relative to the pristine sensor due to the release of a greater number of electrons upon interaction with the adsorbed gas molecules. The oxygen-treated sensor showed a higher response relative to other gas sensors; however, it showed weak selectivity. The non-stoichiometry was the highest in the case of the oxygen-plasma-treated sensor, where the sensitivity was maximum. At RT, the hydrogen-plasma-treated sensor was highly selective to CO gas, while the nitrogen-treated sensor manifested a moderate response to all the gases, without selectivity. Also the argon-plasma-treated sensor did not show noticeable sensitivity to any gas.

Nanowires (NWs) are among the most popular morphologies for gas sensing applications thanks to their high surface area and numerous adsorption sites for gas adsorption. In this context, Pan et al. [101] synthesized SnO_2_ NWs through a CVD method and then used O_2_/Ar plasma to change the compositions to be more non-stoichiometric. The plasma power was varied between 10 and 80 W, while the plasma duration was fixed to 240 s. The samples exposed to plasma under 10, 20, and 40 W had some amounts of SnO, Sn_2_O_3_, and Sn_3_O_4_ phases due to the gradual reduction of tetragonal SnO_2_ and removing of oxygen atoms from SnO_2_. Also, the further increase in plasma power (80 W) resulted in extensive reduction of SnO_2_ to metallic Sn, resulting in poor sensing performance. Among the different gas sensors, the sensor treated with a power of 40 W revealed an enhanced response to ethanol gas, thanks to the co-existence of SnO_2_-Sn_3_O_4_ phases, in which potential barriers at interfaces acted as powerful sources of resistance modulation, in addition to the high surface area thanks to NW morphology and the presence of oxygen vacancies.

In another study, Huang et al. [102] synthesized SnO_2_ thin films using plasma-enhanced CVD (PECVD) and then exposed it to oxygen plasma for 20 min. The pristine sensor manifested a low response of 3.9 to 1000 ppm CO at 330 °C. Also, the plasma-treated sensor showed the highest response of 31.7 to the same gas concentrations at 250 °C. Interestingly, SnO_2_ nanorods (NRs) were grown on SnO_2_ thin films after the plasma treatment by sputtering followed by a redeposition mechanism. In fact, the films were sputtered by the bombardment of heavy ions in the plasma, and then SnO_2_ NRs were grown by the sputtering, redeposition, and rearrangement on the films. Hence, the surface area was significantly increased relative to the pristine SnO_2_ thin film due to the presence of both the 1D NRs and 2D thin film. Accordingly, numerous adsorption sites were available for gas molecules, resulting in a boosted sensing response.

Huang et al. [103] synthesized SnO_2_ nanocolumn arrays with aspect ratios of 20 using liquid immersion PECVD, and the impacts of thermal annealing (600 °C/2 h) and O_2_ plasma treatment on the sensing response toward CO and H_2_ gases were investigated. The response of the pristine sensor to 1000 ppm H_2_ at 400 °C was 17, which was higher than the response to CO gas. Based on a compositional analysis, some residual carbon species remained on the pristine sensor, decreasing its sensing performance. After thermal annealing, the response was increased due to the removal of carbon impurities. Also, after plasma treatment for 40 min, the sensing response to both 1000 ppm CO and H_2_ increased around seven times. The compositional analysis demonstrated that the amount of surface oxygen species significantly increased due to chemisorbed oxygen species on the surface during the plasma treatment, which reacted with target gases to release electrons on the sensor surface.

**Figure 10:**
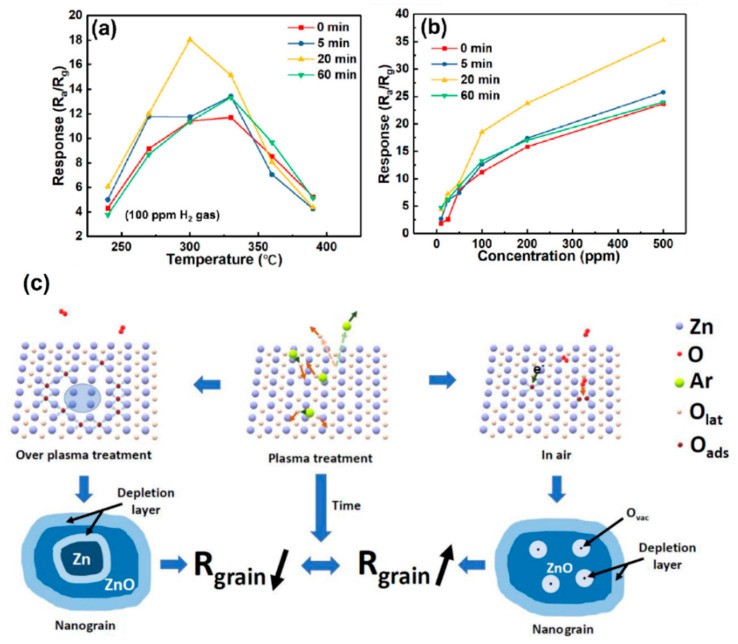
(**a**) Response to H_2_ gas versus temperature and (**b**) calibration curves of plasma-treated gas sensors. (**c**) Mechanism of plasma treatment on a ZnO nanograin [104]. With permission from Elsevier. Copyright (2020).

Hu et al. [104] synthesized ZnO-SnO_2_ heterojunction NFs (200–500 nm) using electrospinning followed by Ar plasma exposure for 5, 20, and 60 min. Overall, all sensors treated with plasma exhibited higher responses than the pristine sensor. Also, at 300 °C, the sensor exposed to plasma for 20 min revealed a response of 18 to 100 ppm H_2_ gas (Figure 10a,b).

Based on an XPS analysis, the amount of adsorbed oxygen species was highest in the optimal sensor. When the plasma was exposed to ZnO, some Zn-O bonds were broken, resulting in the formation of oxygen vacancies. Then, oxygen molecules from the air were adsorbed on the oxygen vacancy sites, and thanks to the highly electrophilic nature of oxygen, they abstracted the electrons from the conduction band of ZnO, leading to the expansion of EDL relative to the pristine ZnO and an increase in resistance. Excess plasma exposure led to the reduction of ZnO to Zn, reducing the overall resistance (Figure 10c). In the case of the optimal gas sensor, plasma exposure caused the formation of EDL with high thickness, and when the sensor was exposed to gas, the release of electrons significantly modulated the sensor resistance. Furthermore, heterojunctions were formed between ZnO and SnO_2_, acting as resistance sources for the gas sensor.

In another study [105], SnO_2_/In_2_O_3_ composite NFs were produced using electrospinning, and then they were exposed to oxygen plasma for 30 min. After plasma exposure, the morphology of SnO_2_ changed to nanoneedles, while that of In_2_O_3_ changed to nanotapers. The surface area before the plasma treatment was 16.5 m^2^/g, and after plasma exposure it increased to 31 m^2^/g. This was due to the fact that the surface was rough and porous after plasma exposure. While the pristine sensor showed a response of 8 to 10 ppm formaldehyde at 375 °C, the response of the plasma-treated sensor was 14 to the same gas concentration at 290 °C. Furthermore, this selective response was related to the small bond dissociation energy of H-CHO, where it was easily broken and reacted with adsorbed oxygen species, releasing electrons on the sensor surface. Due to the plasma treatment, more oxygen species were adsorbed on the surface of sensor, leading to more sensing reactions with formaldehyde gas. In another similar study performed by the same group [106], SnO_2_ NFs revealed an enhanced response to HCHO gas after oxygen plasma treatment. The response of pristine SnO_2_ NFs was only 4.5 to 100 ppm HCHO at 300 °C, while after plasma treatment it was increased to 6.9 at 200 °C.

### 4.5. Plasma-Treated In_2_O_3_ Gas Sensors

One of main shortages of metal oxide gas sensors is humidity interference, which limits their applications in humid environments [107]. Hence, the development of anti-humidity gas sensors is vital. Du et al. [108] synthesized In_2_O_3_ by roasting In_2_SO_4_ at 550 °C, and then fluorocarbon (CF) was grafted onto it by the RF magnetron sputtering technique. The surface of the CF-In_2_O_3_ was evenly wrapped by CF layers with thicknesses of ~2 nm. The In_2_O_3_ film exhibited a low WCA of ∼16° and the CF-In_2_O_3_ film showed a hydrophobic nature with a large WCA of ∼137° thanks to the presence of low-energy CF on the surface of the In_2_O_3_. The In_2_O_3_ recorded a response of ∼18 to 1 ppm NO_2_ gas at 200 °C. However, the CF-In_2_O_3_ sensor revealed a lower response of 13 at an optimal temperature of 100 °C due to the covering of CF on the surface of the In_2_O_3_ with lower sensing properties relative to In_2_O_3_. In the presence of 92% relative humidity, the response of the CF-In_2_O_3_ was not significantly decreased, demonstrating the anti-humidity properties of CF-In_2_O_3_. However, the response of the In_2_O_3_ dramatically decreased. Two reasons can account for the humidity-resistant nature of the optimal sensor: (i) the hydrophobic CF layer absorbed a sufficient amount of H_2_O molecules to increase the electron concentration, and hence more NO_2_ molecules were adsorbed; (ii) the hydrophobic CF layer suppressed the reaction between NO_2_ with H_2_O molecules, and therefore the concentration of the adsorbed and reacted NO_2_ gas molecules on the surface of the sensor did not change.

In another study, Du et al. [109] synthesized In_2_O_3_ NFs and then exposed them to hydrogen and oxygen plasma for 30 min. The surface of the oxygen-plasma-treated In_2_O_3_ was rougher, and the diameters of the NFs were thicker than those of hydrogen plasma In_2_O_3_. However, the diameters of the nanograins on the surface of the NFs were smaller in the case of the oxygen-plasma-treated sample. Also, the surface areas of the pristine, oxygen-, and hydrogen-plasma-treated samples were 18, 32, and 29 m^2^/g, respectively. Thus, the surface area was increased thanks to the formation of many new small pores on the surface of the In_2_O_3_ NFs. Based on an XPS study, the oxygen content was greatly increased by the oxygen plasma, which is vital for sensing reactions with acetone gas. As expected, the sensor exposed to oxygen plasma revealed the largest response of 37 to 500 ppm acetone at 275 °C. The high surface area and the presence of a large amount of adsorbed oxygen species contributed to the enhanced sensing response to acetone.

### 4.6. Other Plasma-Treated Gas Sensors

**Figure 11:**
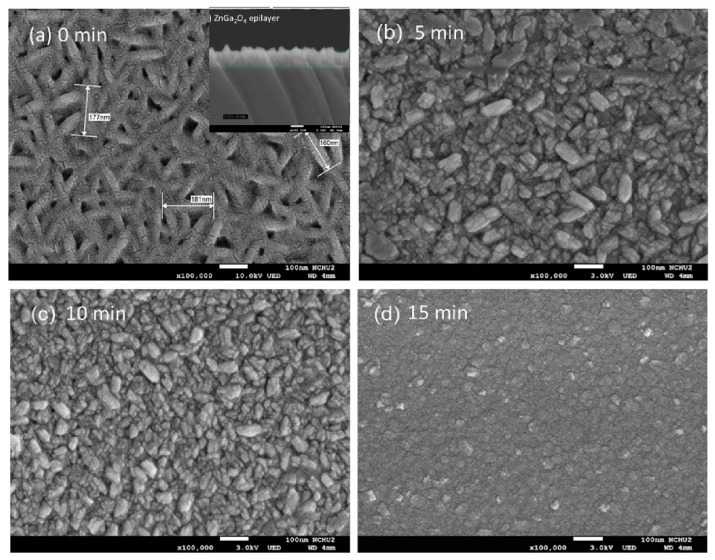
SEM micrographs of ZnGa_2_O_4_ epilayer: (**a**) before and after Ar plasma treatment and (**b**) 5, (**c**) 10, and (**d**) 15 min [111]. With permission from Elsevier. Copyright (2023).

ZnGa_2_O_4_ is a semiconducting material (5.1 eV) with features like the ease of fabrication, low cost, and high stability [110]. Chang et al. [111] synthesized a ZnGa_2_O_4_ epilayer (125 nm thick) on a sapphire substrate using a metal–organic CVD technique. Then, Ar plasma was applied for 5, 10, and 15 min on it. Spindle nanostructures changed to smaller sizes and near-spherical particles after Ar plasma treatment for 15 min due to heavy Ar plasma bombardment and the coalescence of nanostructures (Figure 11a–d). Furthermore, the Ar plasma treatment introduced Ar atoms, radicals, and ions on the epilayer surface, resulting in chemical changes after the plasma treatment.

Among e different sensors, the sensor treated with plasma for 10 min revealed an enhanced response at 300 °C with a response of 1300% to 5 ppm NO gas. The main reasons for sensing enhancement were related to the higher surface area and the presence of more oxygen dangling bonds, leading to an increase in the reactions with NO gas. Also, based on DFT calculations, ZnGa_2_O_4_ with surface oxygen groups had a greater tendency to adsorb NO molecules.

Polypyrrole (PPy) as a CP is a promising sensing material thanks to its high conductance, simple preparation methods, high sensitivity, and possibility of RT operation [112]. Similar to metal oxides, plasma treatment on CPs can increase their gas sensing performance [113]. Zhang et al. [114] applied hydrogen and oxygen plasma on PPy for 20 min and investigated the response to various gases. At 25 °C, the response of the hydrogen-treated sensor to 50 ppm NO_2_ gas was 6, which was 1.6 and 1.2 times higher than that of the pristine and oxygen-treated sensors, respectively. Based on DFT calculations, the adsorption energy of NO_2_ on the hydrogen-treated sensor was significantly higher (−1.72 eV) than that on the pristine (−0.58 eV) and oxygen-treated (−0.69 eV) sensors, respectively. This implied that the hydrogen plasma treatment was more efficient for NO_2_ gas adsorption enhancement. In addition, the increase in surface area by the formation of pores after plasma exposure contributed to the sensing improvement. In another study related to oxygen-plasma-treated PANI, the response to hydrogen at RT was significantly improved relative to the pristine sensor [115].

MXenes are a new category of 2D materials with high conductivity, a large surface area, and tunable band gaps [116]. They have a general formula of M_n+1_X_n_T_x_, in which A is a transition metal, X is C/or N, and T_x_ shows the surface functional groups. They are synthesized from their parent MAX phases, which can be represented as M_n+1_A_n_X, where A is an element in group IIIA or group IVA [117,118]. In this context, Wang et al. [119] synthesized Ti_3_C_2_T*_x_* MXene via liquid exfoliation and subsequently exposed it to oxygen plasma treatment. The sensor exhibited a response of 13.8% to 10 ppm NO_2_ gas at RT. The enhanced sensing performance was related to the presence of numerous oxygen surface functional groups as a result of the plasma treatment.

Transition dichalcogenides are 2D semiconductors with high conductivity and large surface areas. They have a general formula of MX_2_, in which M is a transition metal and X is a chalcogenide such as S, Se, or Te [120,121]. Seo et al. [122] applied Ar plasma treatment on MoS_2_ NSs for 2 s. As a result of plasma exposure, sulfur vacancies were created on the MoS_2_. Then, it was exposed to a 3-mercaptopropionic acid (MPA) solution to form coordinate bonds between the HS groups in MPA and sulfur vacancies. Based on an XPS study, the pristine MoS_2_ exhibited an ideal S/Mo ratio of 1.91, while after plasma exposure it was decreased to 1.51, indicating sulfur vacancy formation. Based on NH_3_ gas sensing studies, the pristine sensor revealed a response of 1.25 to 130 ppm NH_3_ gas at RT, and after treatment by plasma and MPA, the response increased to 4.45. The boosted sensing capability was related to the presence of oxygen and carboxyl groups (−COO) on the surface of the sensor.

Table 1 summarizes the gas sensing properties of plasma-treated gas sensors. Overall, plasma-treated gas sensors have been successfully used for the detection of various toxic gases.

## 5. Conclusions and Outlooks

We reviewed the effect of plasma treatment on the gas sensing characteristics of gas sensors. In general, plasma exposure affects the amount of oxygen species on the sensor surface, and since the oxygen ions are highly required for gas sensing reactions, plasma treatment significantly affects the gas sensing characteristics of resistive sensors through modulation of the amount of oxygen ions. Generally, oxygen plasma causes the addition of surface oxygen functional groups on the sensor surface, and hence, the reactions between adsorbed gases with oxygen increase, leading to a higher sensing performance relative to pristine sensors. Also, exposure to other plasma atmospheres such as Ar or He causes the generation of oxygen defects, which act as favorable sites for oxygen adsorption and accordingly contribute to the enhanced sensing performance. Overall, plasma treatment can cause morphology changes when its power and treatment time are sufficiently high. Also, it causes changes in the amount of oxygen vacancies and adsorbed oxygen species. In some cases, it can add new functional groups on the sensor surface, which act as adsorption sites for gas molecules. Thus, when plasma treatment conditions such as plasma type, time, and power are optimized, it is expected that the sensing properties such as sensitivity, selectivity, and working temperature improve relative to pristine sensors.

Regardless of the type of plasma used, both plasma power and plasma exposure times should be optimized to achieve the highest gas sensing performance. However, in most cases, the focus is on the optimization of plasma time rather than plasma power. Thus, this aspect needs to be more explored in future studies. Different sensing materials such as metal oxides, TMDs, MXenes, CNTs, graphene, and CPs have been subjected to plasma treatment. In this regard, the combination of plasma exposure with other high irradiation techniques such as ion beams, electron beams, and gamma rays can lead to interesting results. Thus, future research directions on plasma-treated gas sensors can be summarized as follows: (i) the development of cheap plasma treatment devices with high availability across the world; (ii) the study of the optimal plasma power and time for various gas sensing materials; (iii) the study of various sensor parameters such as stability, response in humid environments, and reproducibility; and (iv) the reduction in sensing temperature on plasma-treated gas sensors by operation of the sensor in self-heating mode or under UV light illumination.

## Figures and Tables

**Table 1:** Gas sensing properties of plasma-treated gas sensors.

Sensing Material	Synthesis Method	Plasma Condition	Gas	Conc. (ppm)	T (°C)	Response (R_a_/R_g_) or (R_g_/R_a_) or [(R_a_ − R_g_)/R_a_] × 100	Ref.
MWCNTs	CVD	O_2_/C_2_H_2_(CO)_2_O	NH_3_	500	RT	31.4%	[57]
MWCNTs	CVD	Ar	Ethanol	100	65	170%	[60]
SWCNTs	CVD	N_2_	NO_2_	50	RT	121%	[62]
SWCNTs	CVD	N_2_	NH_3_	50	RT	36%	[62]
Graphene	CVD	CF_6_ for 20 s	NH_3_	100	RT	3.8%	[73]
Graphene	CVD	O_3_ for 70 s	NO_2_	10	RT	19.7%	[74]
ZnO thin films	Sol–gel spin coating	O_2_ for 8 min	NH_3_	50	RT	65%	[85]
ZnO NRs	Hydrothermal	O_2_ for 60 s	NMP	100	210	198	[86]
ZnO film	ALD	Ar	TEA	100	250	21.6	[89]
SnO_2_ NSs	Hydrothermal	Ar for 7 min	HCHO	10	270	25	[96]
Pd-SnO_2_ NFs	Electrospinning	Ar for 60 s	H_2_	500	130	53	[99]
SnO_2_ thin film	Plasma enhance CVD	O_2_ for 20 min	CO	1000	250	31.7	[102]
SnO_2_ nanocolumn arrays	Liquid immersion PECVD	O_2_	H_2_	1000	400	17	[103]
ZnO-SnO_2_ heterojunction NFs	Electrospinning	Ar for 20 min	H_2_	100	300	18	[104]
SnO_2_/In_2_O_3_ composite NFs	Electrospinning	O_2_ for 30 min	HCHO	100	290	14	[105]
SnO_2_ NFs	Electrospinning	O_2_	HCHO	100	200	6.9	[106]
In_2_O_3_	Roasting of In_2_SO_4_	Fluorocarbon CF	NO_2_	1	100	13	[108]
In_2_O_3_ NFs	Electrospinning	O_2_	C_3_H_6_O	500	275	37	[109]
ZnGa_2_O_4_	MOCVD technique	Ar for 10 min	NO_2_	5	300	1300%	[111]
PPy	Polymerization	O_2_ for 20 min	NO_2_	50	25	6	[114]
Ti_3_C_2_T*_x_* MXene	Liquid exfoliation	O_2_	NO_2_	10	25	13.8%	[119]
MoS_2_	CVD	Ar for 2 s	NH_3_	130	25	1.25	[122]

## References

[1] Vos T., Lim S.S., Abbafati C., Abbas K.M., Abbasi M., Abbasifard M., Abbasi-Kangevari M., Abbastabar H., Abd-Allah F., Abdelalim A. (2020). Global Burden of 369 Diseases and Injuries in 204 Countries and Territories, 1990–2019: A Systematic Analysis for the Global Burden of Disease Study 2019. Lancet.

[2] Le Quilliec E., Fundere A., Al-U’datt D.G.F., Hiram R. (2023). Pollutants, Including Organophosphorus and Organochloride Pesticides, May Increase the Risk of Cardiac Remodeling and Atrial Fibrillation: A Narrative Review. Biomedicines.

[3] Gal O., Eitan O., Rachum A., Weinberg M., Zigdon D., Assa R., Price C., Yovel Y. (2025). Air Pollution Likely Reduces Hemoglobin Levels in Urban Fruit Bats. iScience.

[4] Manisalidis I., Stavropoulou E., Stavropoulos A., Bezirtzoglou E. (2020). Environmental and Health Impacts of Air Pollution: A Review. Front. Public Health.

[5] Hong C., Zhong R., Xu M., He P., Mo H., Qin Y., Shi D., Chen X., He K., Zhang Q. (2025). Interactions Among Food Systems, Climate Change, and Air Pollution: A Review. Engineering.

[6] Bell J.N.B., Honour S.L., Power S.A. (2011). Effects of Vehicle Exhaust Emissions on Urban Wild Plant Species. Environ. Pollut..

[7] Gao Q., Jiang B., Tong M., Zuo H., Cheng C., Zhang Y., Song S., Lu L., Li X. (2025). Effects and Interaction of Humidex and Air Pollution on Influenza: A National Analysis of 319 Cities in Mainland China. J. Hazard. Mater..

[8] Semczuk-Kaczmarek K., Rys-Czaporowska A., Sierdzinski J., Kaczmarek L.D., Szymanski F.M., Platek A.E. (2022). Association between Air Pollution and COVID-19 Mortality and Morbidity. Intern. Emerg. Med..

[9] Aithal S.S., Sachdeva I., Kurmi O.P. (2023). Air Quality and Respiratory Health in Children. Breathe.

[10] Van Meerbeke S.W., McCarty M., Petrov A.A., Schonffeldt-Guerrero P. (2025). The Impact of Climate, Aeroallergens, Pollution, and Altitude on Exercise-Induced Bronchoconstriction. Immunol. Allergy Clin..

[11] Tiotiu A.I., Novakova P., Nedeva D., Chong-Neto H.J., Novakova S., Steiropoulos P., Kowal K. (2020). Impact of Air Pollution on Asthma Outcomes. Int. J. Environ. Res. Public Health.

[12] Chen K.-C., Tsai S.-W., Shie R.-H., Zeng C., Yang H.-Y. (2022). Indoor Air Pollution Increases the Risk of Lung Cancer. Int. J. Environ. Res. Public Health.

[13] Syama K.P., Blais E., Kumarathasan P. (2025). Maternal Mechanisms in Air Pollution Exposure-Related Adverse Pregnancy Outcomes: A Systematic Review. Sci. Total Environ..

[14] Bayart N.-E., Pereira G., Reid C.M., Nyadanu S.D., Badamdorj O., Lkhagvasuren B., Rumchev K. (2024). Effects of Outdoor Air Pollution on Hospital Admissions from Cardiovascular Diseases in the Capital City of Mongolia. Atmos. Pollut. Res..

[15] Aryal A., Harmon A.C., Dugas T.R. (2021). Particulate Matter Air Pollutants and Cardiovascular Disease: Strategies for Intervention. Pharmacol. Ther..

[16] Glencross D.A., Ho T.-R., Camiña N., Hawrylowicz C.M., Pfeffer P.E. (2020). Air Pollution and Its Effects on the Immune System. Free Radic. Biol. Med..

[17] Sicard P., Agathokleous E., Anenberg S.C., Marco A.D., Paoletti E., Calatayud V. (2023). Trends in Urban Air Pollution over the Last Two Decades: A Global Perspective. Sci. Total Environ..

[18] Li P., Li J., Song S., Chen J., Zhong N., Xie Q., Liu Y., Wan B., He Y., Karimi-Maleh H. (2025). Recent Advances in Optical Gas Sensors for Carbon Dioxide Detection. Measurement.

[19] Gorbova E., Tzorbatzoglou F., Molochas C., Chloros D., Demin A., Tsiakaras P. (2022). Fundamentals and Principles of Solid-State Electrochemical Sensors for High Temperature Gas Detection. Catalysts.

[20] Kumar A., Prajesh R. (2022). The Potential of Acoustic Wave Devices for Gas Sensing Applications. Sens. Actuators A Phys..

[21] El-Muraikhi M.D., Ayesh A.I., Mirzaei A. (2025). Resistive Gas Sensors Based on Inorganic Nanotubes: A Review. J. Alloys Compd..

[22] Mirzaei A., Neri G. (2016). Microwave-Assisted Synthesis of Metal Oxide Nanostructures for Gas Sensing Application: A Review. Sens. Actuators B Chem..

[23] Guo S.-Y., Hou P.-X., Zhang F., Liu C., Cheng H.-M. (2022). Gas Sensors Based on Single-Wall Carbon Nanotubes. Molecules.

[24] Mirzaei A., Bharath S.P., Kim J.-Y., Pawar K.K., Kim H.W., Kim S.S. (2023). N-Doped Graphene and Its Derivatives as Resistive Gas Sensors: An Overview. Chemosensors.

[25] Kiranakumar H.V., Thejas R., Naveen C.S., Khan M.I., Prasanna G.D., Reddy S., Oreijah M., Guedri K., Bafakeeh O.T., Jameel M. (2024). A Review on Electrical and Gas-Sensing Properties of Reduced Graphene Oxide-Metal Oxide Nanocomposites. Biomass Convers. Biorefinery.

[26] Matindoust S., Farzi G., Nejad M.B., Shahrokhabadi M.H. (2021). Polymer-Based Gas Sensors to Detect Meat Spoilage: A Review. React. Funct. Polym..

[27] Hu Y., Zheng W., Fan S., Zhang J., Liu X. (2023). Noble-Transition-Metal Dichalcogenides-Emerging Two-Dimensional Materials for Sensor Applications. Appl. Phys. Rev..

[28] Amiri V., Roshan H., Mirzaei A., Neri G., Ayesh A.I. (2020). Nanostructured Metal Oxide-Based Acetone Gas Sensors: A Review. Sensors.

[29] Bonyani M., Zebarjad S.M., Mirzaei A., Kim T.-U., Kim H.W., Kim S.S. (2024). Electrospun ZnO Hollow Nanofibers Gas Sensors: An Overview. J. Alloys Compd..

[30] El-Muraikhi M.D., Ayesh A.I., Mirzaei A. (2025). Review of Nanostructured Bi_2_O_3_, Bi_2_WO_6_, and BiVO_4_ as Resistive Gas Sensors. Surf. Interfaces.

[31] El-Muraikhi M.D., Mirzaei A., Ayesh A.I. (2024). Nanostructured Nb_2_O_5_ as Chemiresistive Gas Sensors. Ceram. Int..

[32] Zappa D., Galstyan V., Kaur N., Arachchige H.M.M.M., Sisman O., Comini E. (2018). “Metal Oxide -Based Heterostructures for Gas Sensors”—A Review. Anal. Chim. Acta.

[33] Degler D., Weimar U., Barsan N. (2019). Current Understanding of the Fundamental Mechanisms of Doped and Loaded Semiconducting Metal-Oxide-Based Gas Sensing Materials. ACS Sens..

[34] Zhu L.-Y., Ou L.-X., Mao L.-W., Wu X.-Y., Liu Y.-P., Lu H.-L. (2023). Advances in Noble Metal-Decorated Metal Oxide Nanomaterials for Chemiresistive Gas Sensors: Overview. Nano-Micro Lett..

[35] Mirzaei A., Ansari H.R., Shahbaz M., Kim J.-Y., Kim H.W., Kim S.S. (2022). Metal Oxide Semiconductor Nanostructure Gas Sensors with Different Morphologies. Chemosensors.

[36] Liu M., Song P., Wang Q., Yan M. (2024). CsPbBr3 Quantum Dot Modified In_2_O_3_ Nanofibers for Effective Detection of Ppb-Level HCHO at Room Temperature under UV Illumination. ACS Sens..

[37] Majhi S.M., Mirzaei A., Navale S., Kim H.W., Kim S.S. (2021). Boosting the Sensing Properties of Resistive-Based Gas Sensors by Irradiation Techniques: A Review. Nanoscale.

[38] Kim J.H., Lee M.A., Han G.J., Cho B.H. (2013). Plasma in Dentistry: A Review of Basic Concepts and Applications in Dentistry. Acta Odontol. Scand..

[39] Bayati M., Lund M.N., Tiwari B.K., Poojary M.M. (2024). Chemical and Physical Changes Induced by Cold Plasma Treatment of Foods: A Critical Review. Compr. Rev. Food Sci. Food Saf..

[40] Bezerra J.d.A., Lamarão C.V., Sanches E.A., Rodrigues S., Fernandes F.A.N., Ramos G.L.P.A., Esmerino E.A., Cruz A.G., Campelo P.H. (2023). Cold Plasma as a Pre-Treatment for Processing Improvement in Food: A Review. Food Res. Int..

[41] Nimbekar A.A., Deshmukh R.R. (2022). Plasma Surface Modification of Flexible Substrates to Improve Grafting for Various Gas Sensing Applications: A Review. IEEE Trans. Plasma Sci..

[42] Alemán C., Fabregat G., Armelin E., Buendía J.J., Llorca J. (2018). Plasma Surface Modification of Polymers for Sensor Applications. J. Mater. Chem. B.

[43] Saka C. (2017). Overview on the Surface Functionalization Mechanism and Determination of Surface Functional Groups of Plasma Treated Carbon Nanotubes. Crit. Rev. Anal. Chem..

[44] Leghrib R., Felten A., Demoisson F., Reniers F., Pireaux J.-J., Llobet E. (2010). Room-Temperature, Selective Detection of Benzene at Trace Levels Using Plasma-Treated Metal-Decorated Multiwalled Carbon Nanotubes. Carbon.

[45] Ambardekar V., Sahoo S., Srivastava D.K., Majumder S.B., Bandyopadhyay P.P. (2021). Plasma Sprayed CuO Coatings for Gas Sensing and Catalytic Conversion Applications. Sens. Actuators B Chem..

[46] Ambardekar V., Bandyopadhyay P.P., Majumder S.B. (2021). Plasma Sprayed Copper Oxide Sensor for Selective Sensing of Carbon Monoxide. Mater. Chem. Phys..

[47] Choi M.S., Bang J.H., Mirzaei A., Na H.G., Kwon Y.J., Kang S.Y., Choi S.-W., Kim S.S., Kim H.W. (2018). Dual Sensitization of MWCNTs by Co-Decoration with p- and n-Type Metal Oxide Nanoparticles. Sens. Actuators B Chem..

[48] Kwon Y.J., Mirzaei A., Kang S.Y., Choi M.S., Bang J.H., Kim S.S., Kim H.W. (2017). Synthesis, Characterization and Gas Sensing Properties of ZnO-Decorated MWCNTs. Appl. Surf. Sci..

[49] Lavagna L., Nisticò R., Musso S., Pavese M. (2021). Functionalization as a Way to Enhance Dispersion of Carbon Nanotubes in Matrices: A Review. Mater. Today Chem..

[50] Jian J., Guo X., Lin L., Cai Q., Cheng J., Li J. (2013). Gas-Sensing Characteristics of Dielectrophoretically Assembled Composite Film of Oxygen Plasma-Treated SWCNTs and PEDOT/PSS Polymer. Sens. Actuators B Chem..

[51] Leghrib R., Pavelko R., Felten A., Vasiliev A., Cané C., Gràcia I., Pireaux J.-J., Llobet E. (2010). Gas Sensors Based on Multiwall Carbon Nanotubes Decorated with Tin Oxide Nanoclusters. Sens. Actuators B Chem..

[52] Min S., Kim J., Park C., Jin J.-H., Min N.K. (2017). Long-Term Stability of Superhydrophilic Oxygen Plasma-Modified Single-Walled Carbon Nanotube Network Surfaces and the Influence on Ammonia Gas Detection. Appl. Surf. Sci..

[53] Yeo S., Choi C., Woong Jang C., Lee S., Min Jhon Y. (2013). Sensitivity Enhancement of Carbon Nanotube Based Ammonium Ion Sensors through Surface Modification by Using Oxygen Plasma Treatment. Appl. Phys. Lett..

[54] Zhang X., Wu X., Yang B., Xiao H. (2015). Enhancement of Gas Sensing Characteristics of Multiwalled Carbon Nanotubes by CF4 Plasma Treatment for SF6 Decomposition Component Detection. J. Nanomater..

[55] Clément P., Ramos A., Lazaro A., Molina-Luna L., Bittencourt C., Girbau D., Llobet E. (2015). Oxygen Plasma Treated Carbon Nanotubes for the Wireless Monitoring of Nitrogen Dioxide Levels. Sens. Actuators B Chem..

[56] Zhang X., Yang B., Wang X., Luo C. (2012). Effect of Plasma Treatment on Multi-Walled Carbon Nanotubes for the Detection of H_2_S and SO_2_. Sensors.

[57] Bannov A.G., Jašek O., Manakhov A., Márik M., Nečas D., Zajíčková L. (2017). High-Performance Ammonia Gas Sensors Based on Plasma Treated Carbon Nanostructures. IEEE Sens. J..

[58] Bannov A.G., Manakhov A.M., Shtansky D.V. (2022). Plasma Functionalization of Multi-Walled Carbon Nanotubes for Ammonia Gas Sensors. Materials.

[59] Dong K.-Y., Ham D.-J., Kang B.H., Lee K., Choi J., Lee J.-W., Choi H.H., Ju B.-K. (2012). Effect of Plasma Treatment on the Gas Sensor with Single-Walled Carbon Nanotube Paste. Talanta.

[60] Santhosh N.M., Vasudevan A., Jurov A., Korent A., Slobodian P., Zavašnik J., Cvelbar U. (2020). Improving Sensing Properties of Entangled Carbon Nanotube-Based Gas Sensors by Atmospheric Plasma Surface Treatment. Microelectron. Eng..

[61] Ham S.W., Hong H.P., Kim J.H., Min S.J., Min N.K. (2014). Effect of Oxygen Plasma Treatment on Carbon Nanotube-Based Sensors. J. Nanosci. Nanotechnol..

[62] Zamansky K.K., Osipova A.A., Fedorov F.S., Kopylova D.S., Shunaev V., Alekseeva A., Glukhova O.E., Nasibulin A.G. (2023). Sensitivity Enhancement of SWCNT Gas Sensors by Nitrogen Plasma Treatment. Appl. Surf. Sci..

[63] Ham S.W., Hong H.P., Kim J.W., Kim J.H., Kim K.B., Park C.W., Min N.K. (2015). Comparison of Gas Sensors Based on Oxygen Plasma-Treated Carbon Nanotube Network Films with Different Semiconducting Contents. J. Electron. Mater..

[64] Farea M.A., Mohammed H.Y., Shirsat S.M., Sayyad P.W., Ingle N.N., Al-Gahouari T., Mahadik M.M., Bodkhe G.A., Shirsat M.D. (2021). Hazardous Gases Sensors Based on Conducting Polymer Composites: Review. Chem. Phys. Lett..

[65] Liu X., Zheng W., Kumar R., Kumar M., Zhang J. (2022). Conducting Polymer-Based Nanostructures for Gas Sensors. Coord. Chem. Rev..

[66] Guo X., Jian J., Lin L., Zhu H., Zhu S. (2013). O_2_ Plasma-Functionalized SWCNTs and PEDOT/PSS Composite Film Assembled by Dielectrophoresis for Ultrasensitive Trimethylamine Gas Sensor. Analyst.

[67] Yoo K.-P., Kwon K.-H., Min N.-K., Lee M.J., Lee C.J. (2009). Effects of O_2_ Plasma Treatment on NH3 Sensing Characteristics of Multiwall Carbon Nanotube/Polyaniline Composite Films. Sens. Actuators B Chem..

[68] Kim J.H., Song M.-J., Kim K.B., Jin J.-H., Min N.K. (2017). Evaluation of Surface Cleaning Procedures in Terms of Gas Sensing Properties of Spray-Deposited CNT Film: Thermal- and O_2_ Plasma Treatments. Sensors.

[69] Zhao W., Fam D.W.H., Yin Z., Sun T., Tan H.T., Liu W., Tok A.I.Y., Boey Y.C.F., Zhang H., Hng H.H. (2012). A Carbon Monoxide Gas Sensor Using Oxygen Plasma Modified Carbon Nanotubes. Nanotechnology.

[70] Schedin F., Geim A.K., Morozov S.V., Hill E.W., Blake P., Katsnelson M.I., Novoselov K.S. (2007). Detection of Individual Gas Molecules Adsorbed on Graphene. Nat. Mater..

[71] Wu H., Bu X., Deng M., Chen G., Zhang G., Li X., Wang X., Liu W. (2019). A Gas Sensing Channel Composited with Pristine and Oxygen Plasma-Treated Graphene. Sensors.

[72] Capote Mastrapa G., Freire F.L. (2019). Plasma-Treated CVD Graphene Gas Sensor Performance in Environmental Condition: The Role of Defects on Sensitivity. J. Sens..

[73] Zhang H., Fan L., Dong H., Zhang P., Nie K., Zhong J., Li Y., Guo J., Sun X. (2016). Spectroscopic Investigation of Plasma-Fluorinated Monolayer Graphene and Application for Gas Sensing. ACS Appl. Mater. Interfaces.

[74] Chung M.G., Kim D.H., Lee H.M., Kim T., Choi J.H., Seo D.k., Yoo J.-B., Hong S.-H., Kang T.J., Kim Y.H. (2012). Highly Sensitive NO_2_ Gas Sensor Based on Ozone Treated Graphene. Sens. Actuators B Chem..

[75] Zhang C., Xu K., Liu K., Xu J., Zheng Z. (2022). Metal Oxide Resistive Sensors for Carbon Dioxide Detection. Coord. Chem. Rev..

[76] Casanova-Chafer J., Garcia-Aboal R., Llobet E., Atienzar P. (2024). Enhanced CO_2_ Sensing by Oxygen Plasma-Treated Perovskite–Graphene Nanocomposites. ACS Sens..

[77] Chowdhury I., Duch M.C., Mansukhani N.D., Hersam M.C., Bouchard D. (2013). Colloidal Properties and Stability of Graphene Oxide Nanomaterials in the Aquatic Environment. Environ. Sci. Technol..

[78] Dimiev A.M., Tour J.M. (2014). Mechanism of Graphene Oxide Formation. ACS Nano.

[79] Thangamani G.J., Deshmukh K., Kovářík T., Nambiraj N.A., Ponnamma D., Sadasivuni K.K., Khalil H.P.S.A., Pasha S.K.K. (2021). Graphene Oxide Nanocomposites Based Room Temperature Gas Sensors: A Review. Chemosphere.

[80] Hafiz S.M., Ritikos R., Whitcher T.J., Razib N.M., Bien D.C.S., Chanlek N., Nakajima H., Saisopa T., Songsiriritthigul P., Huang N.M. (2014). A Practical Carbon Dioxide Gas Sensor Using Room-Temperature Hydrogen Plasma Reduced Graphene Oxide. Sens. Actuators B Chem..

[81] Hamzaj A.K., Donà E., Santhosh N.M., Shvalya V., Košiček M., Cvelbar U. (2024). Plasma-Modification of Graphene Oxide for Advanced Ammonia Sensing. Appl. Surf. Sci..

[82] Gruber D., Kraus F., Müller J. (2003). A Novel Gas Sensor Design Based on CH_4_/H_2_/H_2_O Plasma Etched ZnO Thin Films. Sens. Actuators B Chem..

[83] Law J.B.K., Thong J.T.L. (2008). Improving the NH3 Gas Sensitivity of ZnO Nanowire Sensors by Reducing the Carrier Concentration. Nanotechnology.

[84] Wang G., Chen T., Guo L., Wang W., Wang H., Wang Y., Zeng H., Liu X., Wang J., Yang Y. (2023). Highly Response Gas Sensor Based the Au-ZnO Films Processed by Combining Magnetron Sputtering and Ar Plasma Treatment. Phys. Scr..

[85] Hou Y., Jayatissa A.H. (2014). Enhancement of Gas Sensor Response of Nanocrystalline Zinc Oxide for Ammonia by Plasma Treatment. Appl. Surf. Sci..

[86] Gui Y., Zhao S., Tian K., Wu J., Guo H., Qin X., Qin X., Guo D., Zheng G., Guo Y. (2024). Oxygen Plasma Treatment to Enhance the Gas-Sensing Performance of ZnO to N-Methyl Pyrrolidone: Experimental and Computational Study. Ceram. Int..

[87] George S.M. (2010). Atomic Layer Deposition: An Overview. Chem. Rev..

[88] Xu Y., Zheng W., Liu X., Zhang L., Zheng L., Yang C., Pinna N., Zhang J. (2020). Platinum Single Atoms on Tin Oxide Ultrathin Films for Extremely Sensitive Gas Detection. Mater. Horiz..

[89] Li Z., Liu X., Zhou M., Zhang S., Cao S., Lei G., Lou C., Zhang J. (2021). Plasma-Induced Oxygen Vacancies Enabled Ultrathin ZnO Films for Highly Sensitive Detection of Triethylamine. J. Hazard. Mater..

[90] Das S., Jayaraman V. (2014). SnO_2_: A Comprehensive Review on Structures and Gas Sensors. Prog. Mater. Sci..

[91] Masuda Y. (2022). Recent Advances in SnO_2_ Nanostructure Based Gas Sensors. Sens. Actuators B Chem..

[92] Hiyoto K.A.M., Fisher E.R. (2020). Utilizing Plasma Modified SnO_2_ Paper Gas Sensors to Better Understand Gas-Surface Interactions at Low Temperatures. J. Vac. Sci. Technol. A.

[93] Stuckert E.P., Miller C.J., Fisher E.R. (2017). The Effect of Ar/O_2_ and H_2_O Plasma Treatment of SnO_2_ Nanoparticles and Nanowires on Carbon Monoxide and Benzene Detection. ACS Appl. Mater. Interfaces.

[94] Srivastava R., Dwivedi R., Srivastava S.K. (1998). Effect of Oxygen and Hydrogen Plasma Treatment on the Room Temperature Sensitivity of SnO_2_ Gas Sensors. Microelectron. J..

[95] Chaturvedi A., Mishra V.N., Dwivedi R., Srivastava S.K. (1999). Response of Oxygen Plasma-Treated Thick Film Tin Oxide Sensor Array for LPG, CCl_4_, CO and C_3_H_7_OH. Microelectron. J..

[96] Acharyya S., Guha P.K. (2024). Enhanced Formaldehyde Sensing Performance Employing Plasma-Treated Hierarchical SnO_2_ Nanosheets through Oxygen Vacancy Modulation. Appl. Surf. Sci..

[97] Mirzaei A., Yousefi H.R., Falsafi F., Bonyani M., Lee J.-H., Kim J.-H., Kim H.W., Kim S.S. (2019). An Overview on How Pd on Resistive-Based Nanomaterial Gas Sensors Can Enhance Response toward Hydrogen Gas. Int. J. Hydrogen Energy.

[98] Zhu M., Zhang H., Zhang S., Yao H., Shi X., Xu S. (2025). Chemoresistive Gas Sensors Based on Noble-Metal-Decorated Metal Oxide Semiconductors for H2 Detection. Materials.

[99] Hu K., Wang F., Liu H., Li Y., Zeng W. (2020). Enhanced Hydrogen Gas Sensing Properties of Pd-Doped SnO_2_ Nanofibres by Ar Plasma Treatment. Ceram. Int..

[100] Chaturvedi A., Mishra V.N., Dwivedi R., Srivastava S.K. (2000). Selectivity and Sensitivity Studies on Plasma Treated Thick Film Tin Oxide Gas Sensors. Microelectron. J..

[101] Pan J., Ganesan R., Shen H., Mathur S. (2010). Plasma-Modified SnO_2_ Nanowires for Enhanced Gas Sensing. J. Phys. Chem. C.

[102] Huang H., Tan O.K., Lee Y.C., Tran T.D., Tse M.S., Yao X. (2005). Semiconductor Gas Sensor Based on Tin Oxide Nanorods Prepared by Plasma-Enhanced Chemical Vapor Deposition with Postplasma Treatment. Appl. Phys. Lett..

[103] Huang H., Lee Y.C., Chow C.L., Tan O.K., Tse M.S., Guo J., White T. (2009). Plasma Treatment of SnO_2_ Nanocolumn Arrays Deposited by Liquid Injection Plasma-Enhanced Chemical Vapor Deposition for Gas Sensors. Sens. Actuators B Chem..

[104] Hu K., Wang F., Shen Z., Liu H., Zeng W., Wang Y. (2020). Ar Plasma Treatment on ZnO–SnO_2_ Heterojunction Nanofibers and Its Enhancement Mechanism of Hydrogen Gas Sensing. Ceram. Int..

[105] Du H., Wang J., Sun Y., Yao P., Li X., Yu N. (2015). Investigation of Gas Sensing Properties of SnO_2_/In_2_O_3_ Composite Hetero-Nanofibers Treated by Oxygen Plasma. Sens. Actuators B Chem..

[106] Du H.-Y., Wang J., Yu P., Yu N.-S., Sun Y.-H., Tian J.-L. (2014). Investigation of Gas Sensing Materials Tin Oxide Nanofibers Treated by Oxygen Plasma. J. Nanoparticle Res..

[107] Jyothilal H., Shukla G., Walia S., Kundu S., Angappane S. (2020). Humidity Sensing and Breath Analyzing Applications of TiO_2_ Slanted Nanorod Arrays. Sens. Actuators A Phys..

[108] Du B., Qi T., Li J., He Y., Yang X. (2021). Improving Anti-Humidity Property of In_2_O_3_ Based NO_2_ Sensor by Fluorocarbon Plasma Treatment. Sens. Actuators B Chem..

[109] Du H., Wang H., Yao P., Wang J., Sun Y. (2018). In_2_O_3_ Nanofibers Surface Modified by Low-Temperature RF Plasma and Their Gas Sensing Properties. Mater. Chem. Phys..

[110] Wu M.-R., Li W.-Z., Tung C.-Y., Huang C.-Y., Chiang Y.-H., Liu P.-L., Horng R.-H. (2019). NO Gas Sensor Based on ZnGa_2_O_4_ Epilayer Grown by Metalorganic Chemical Vapor Deposition. Sci. Rep..

[111] Chang T.-Y., Singh A.K., Shao J.-H., Huang C.-Y., Shieh J.-M., Wuu D.-S., Liu P.-L., Horng R.-H. (2023). Performance Improvement of MOCVD Grown ZnGa_2_O_4_ Based NO Gas Sensors Using Plasma Surface Treatment. Appl. Surf. Sci..

[112] Das M., Roy S. (2021). Polypyrrole and Associated Hybrid Nanocomposites as Chemiresistive Gas Sensors: A Comprehensive Review. Mater. Sci. Semicond. Process..

[113] Suzuki T., Tanner P., Thiel D.V. (2002). O_2_ Plasma Treated Polyimide-Based Humidity Sensors. Analyst.

[114] Zhang Z., Zhao L., Du H., Chu J. (2023). Improved NO2 Gas-Sensing Performance of PPy by Hydrogen Plasma Treatment: Experimental Study and DFT Verification. Sens. Actuators A Phys..

[115] Kunzo P., Lobotka P., Micusik M., Kovacova E. (2012). Palladium-Free Hydrogen Sensor Based on Oxygen-Plasma-Treated Polyaniline Thin Film. Sens. Actuators B Chem..

[116] Cao W., Nie J., Cao Y., Gao C., Wang M., Wang W., Lu X., Ma X., Zhong P. (2024). A Review of How to Improve Ti_3_C_2_T_x_ MXene Stability. Chem. Eng. J..

[117] Mirzaei A., Kim J.-Y., Kim H.W., Kim S.S. (2024). Resistive Gas Sensors Based on 2D TMDs and MXenes. Acc. Chem. Res..

[118] Mirzaei A., Kim J.-Y., Kim J.H., Nam M.-S., Kim H.W., Kim S.S. (2025). Accordion-like Ti_3_C_2_T_x_ MXene with High Flexibility for NH_3_ Sensing in Self-Heating Mode. Ceram. Int..

[119] Wang Y., Fu J., Xu J., Hu H., Ho D. (2023). Atomic Plasma Grafting: Precise Control of Functional Groups on Ti_3_C_2_T_x_ MXene for Room Temperature Gas Sensors. ACS Appl. Mater. Interfaces.

[120] Ansari H.R., Mirzaei A., Shokrollahi H., Kumar R., Kim J.-Y., Kim H.W., Kumar M., Kim S.S. (2023). Flexible/Wearable Resistive Gas Sensors Based on 2D Materials. J. Mater. Chem. C.

[121] Sharma R., Laishram R., Gupta B.K., Srivastva R., Sinha O.P. (2022). A Review on MX2 (M = Mo, W and X = S, Se) Layered Material for Opto-Electronic Devices. Adv. Nat. Sci. Nanosci. Nanotechnol..

[122] Seo W.S., Kim D.K., Han J.-H., Park K.-B., Ryu S.C., Min N.K., Kim J.H. (2020). Functionalization of Molybdenum Disulfide via Plasma Treatment and 3-Mercaptopropionic Acid for Gas Sensors. Nanomaterials.

